# Muslim communities learning about second-hand smoke (MCLASS): study protocol for a pilot cluster randomised controlled trial

**DOI:** 10.1186/1745-6215-14-295

**Published:** 2013-09-13

**Authors:** Hannah Ainsworth, Sarwat Shah, Faraz Ahmed, Amanda Amos, Ian Cameron, Caroline Fairhurst, Rebecca King, Ghazala Mir, Steve Parrott, Aziz Sheikh, David Torgerson, Heather Thomson, Kamran Siddiqi

**Affiliations:** 1 York Trials Unit, Department of Health Sciences, University of York, York, North Yorkshire YO10 5DD, England; 2 Department of Health Sciences, University of York, York, North Yorkshire YO10 5DD, England; 3 Cambridge Centre for Health Services Research, University of Cambridge, Institute of Public Health, Forvie Site, Robinson Way, Cambridge CB2 0SR, England; 4 UKCTCS, Centre for Population Health Sciences, University of Edinburgh, Medical School, Teviot Place, Edinburgh EH8 9AG, Scotland; 5 Leeds City Council, Civic Hall, Calverley Street, Leeds LS1 1UR, England; 6 Leeds Institute of Health Sciences, University of Leeds, Charles Thackrah Building, 101 Clarendon Road, Leeds LS2 9LJ, England; 7 Allergy & Respiratory Research Group, Centre for Population Health Sciences, University of Edinburgh, Medical School, Teviot Place, Edinburgh EH8 9AG, Scotland

**Keywords:** Bangladeshi, Ethnicity, Faith leaders, Mixed-methods, Muslim, Pakistani, Pilot, Cluster randomised control trial, Second-hand smoking, Smoking

## Abstract

**Background:**

In the UK, 40% of Bangladeshi and 29% of Pakistani men smoke cigarettes regularly compared to the national average of 24%. As a consequence, second-hand smoking is also widespread in their households which is a serious health hazard to non-smokers, especially children. Smoking restrictions in households can help reduce exposure to second-hand smoking. This is a pilot trial of ‘Smoke Free Homes’, an educational programme which has been adapted for use by Muslim faith leaders, in an attempt to find an innovative solution to encourage Pakistani- and Bangladeshi-origin communities to implement smoking restrictions in their homes. The primary objectives for this pilot trial are to establish the feasibility of conducting such an evaluation and provide information to inform the design of a future definitive study.

**Methods/Design:**

This is a pilot cluster randomised controlled trial of ‘Smoke Free Homes’, with an embedded preliminary health economic evaluation and a qualitative analysis. The trial will be carried out in around 14 Islamic religious settings. Equal randomisation will be employed to allocate each cluster to a trial arm. The intervention group will be offered the Smoke Free Homes package (*Smoke Free Homes: a resource for Muslim religious teachers*), trained in its use, and will subsequently implement the package in their religious settings. The remaining clusters will not be offered the package until the completion of the study and will form the control group. At each cluster, we aim to recruit around 50 households with at least one adult resident who smokes tobacco and at least one child or a non-smoking adult. Households will complete a household survey and a non-smoking individual will provide a saliva sample which will be tested for cotinine. All participant outcomes will be measured before and after the intervention period in both arms of the trial. In addition, a purposive sample of participants and religious leaders/teachers will take part in interviews and focus groups.

**Discussion:**

The results of this pilot study will inform the protocol for a definitive trial.

**Trial registration:**

Current Controlled Trials ISRCTN03035510

## Background

Every year an estimated 79,000 adults die due to exposure to second-hand smoke (SHS) from tobacco in European Union countries [1], of which more than 11,000 are from the United Kingdom (UK) [2]. It has been more than six years since the legislation to ban smoking in enclosed public places was implemented in the UK. This has resulted in a welcome decline in SHS exposure and also illustrates the positive role of comprehensive smoke-free legislation. However, it is concerning that certain population subgroups (that is, lower socioeconomic status and certain ethnic groups) with the greatest SHS exposure benefited the least from the change [3]. As expected, living in a home without any smoking restrictions is an important determinant of exposure [4,5]. SHS is associated with development of lung cancer, coronary heart disease, respiratory disease and stroke among adult non-smokers [6-8]. Among children it also increases risk of other lower respiratory illnesses, chronic middle ear disease and sudden infant death syndrome [1,9].

In the UK, an estimated 40% of Bangladeshi and 29% of Pakistani men regularly smoke cigarettes compared to the national average of 24% (these figures are from 2004 but are the latest available broken down by ethinicity) [10]. Regular smoking has also been shown to be common (39%) among 14 to 15 year old boys of Bangladeshi-origin in a London-based survey [10]. SHS is also common in Bangladeshi and Pakistani households. For example, a study in 2008 in a locality in which nearly 50% of the population was of South Asian origin, found that smoking takes place regularly in front of children in 42% (95% confidence interval: 35% to 50%) of all households with at least one smoker [11]. Another study reported higher salivary cotinine among Bangladeshi-origin children in households with smokers compared to children from other ethnic backgrounds [12]. Smoking quit rates are lower than the UK average in these groups despite a high motivation to quit [13]. This may in part be due to a feeling of ‘isolation and marginalisation’ from the existing smoking cessation services and lack of benefit from smoke-free initiatives [14].

Higher susceptibility and earlier onset of cardiovascular diseases (CVD) [15,16] and accumulation of other risk factors (for example, diabetes), result in particularly poor health outcomes among the target communities. At the age of 50, Bangladeshi and Pakistani men without other risk factors have a 13% risk of a CVD event within 10 years compared to 8% in the general UK population [17]. This risk increases to 22% compared to 14% in the general UK smoking population, this being higher than for any other UK ethnic group [17]. Babies born to mothers of Pakistani birth have a higher infant mortality rate (9%) compared to those whose mothers were born in the UK (5%) [18,19]. Moreover, the average birth weight is significantly lower in babies born to mothers of South Asian origin – for both first generation - (7%) and second-generation (12%) migrants – than the national average. These children have higher childhood morbidity than those of White European-origin parents [20]. In addition, there is a higher risk of admission for asthma in children of South Asian origin compared to White populations [21].

A smoke-free home is not just associated with lower levels of SHS, but there is also evidence that suggests a strong link with increased smoking cessation and decreased cigarette consumption amongst adult smokers themselves [22]. Moreover, qualitative studies of smoking cessation behaviour have found that Pakistani and Bangladeshi smokers have high levels of motivation to quit, but that this does not often translate into success, thus, highlighting the need for culturally acceptable and feasible interventions for ethnic minority communities [13,23,24].

Adaptation of health promotion and disease prevention interventions for Bangladeshi- and Pakistani-origin Muslim communities requires an understanding of both ‘surface’ and ‘deep’ dimensions of cultural sensitivities [25]. The ‘surface’ dimension includes superficial structures, such as people, clothing, language and so on, which allows the intervention to be acceptable. Consideration of the ‘deep’ dimensions, such as religious and socio-cultural constructs of the community, helps in making connections with the underlying beliefs, values and structures of communities thereby enhancing salience, acceptability and uptake of the health interventions [26].

There is emerging consensus amongst Muslims on the religious prohibition of the use of tobacco-containing products [27]. Whilst our review of the body of Islamic text/literature found no direct references prohibiting tobacco use (unsurprisingly given the relatively recent emergence of smoking as a social phenomenon), there are several indirect references that are interpreted by several Islamic scholars as a discouragement of its use on the basis of its addictive nature and harm to one’s own and others’ health [27]. Religion is an important determinant of beliefs and attitudes towards smoking in Bangladeshi- and Pakistani-origin Muslim communities [28,29] and influences decision-making about health behaviour. Many believe that smoking is in conflict with Islamic teaching, even if not strictly prohibited [28]. In a study in Pakistan, Imams used Friday sermons to encourage people to implement smoking restrictions at home with a positive effect [30]. This suggests that mosques using their influential status in the Bangladeshi- and Pakistani-origin Muslim communities in the UK could play an important role in shifting social norms around smoking behaviours.

With this in mind, we developed a ‘Smoke Free Homes’ (SFH) package to be used by faith leaders for the benefit of Bangladeshi- and Pakistani-origin Muslim communities. The package was developed in collaboration with faith leaders and mosques, and a feasibility study was conducted in five mosques in Leeds. The SFH activities were highlighted as being acceptable and appropriate given the setting [31]. Whilst this preliminary work suggested the potential for an important impact, the study design precluded reliable inferences to measure its effectiveness and was unable to provide estimates for a definitive trial. We have, therefore, designed this pilot randomised controlled trial (RCT) that will allow us to inform, test and improve the trial protocol and design for a definitive trial.

### Aim

The objective of a definitive trial would be to investigate whether SFH delivered by faith leaders in Islamic religious settings is an effective way of protecting non-smokers by reducing exposure to SHS, as measured by salivary cotinine levels. Secondary aims would be to determine its effectiveness in reducing the uptake of smoking and improving smoking quit rates in Bangladeshi and Pakistani origin Muslim communities in the UK.

This pilot trial (MCLASS: Muslim Communities Learning About Second-hand Smoke) is designed to assess the feasibility of conducting a large definitive trial.

### Research questions

This pilot trial has been designed to answer the following key research questions:

Number of clusters (Islamic religious settings) and the size of each cluster (participants) for the main trial

1. What are the recruitment and attrition rates for clusters and participants?

2. Are clusters and participants willing to be randomised?

3. What is the likely effect size in relation to the primary outcome measure (that is, salivary cotinine)?

4. What is the intra-cluster correlation coefficient (ICC) for the primary outcome among participants?

5. What is the likely potential of contamination between clusters?

Feasibility and resource requirements to deliver the intervention and assess its outcomes

6. What are the costs associated with delivering ‘SFH’ through Islamic religious settings?

7. What is the feasibility and acceptability of measuring the primary outcome (salivary cotinine)?

8. What is the response rate for the household survey to assess smoking behaviour?

9. Are data collection questions to capture health care utilisation acceptable and useful?

Integration of ‘SFH’ in Islamic religious settings

10. What are the facilitators and barriers for integration of ‘SFH’ into Islamic religious settings practice and how might facilitators be enhanced / barriers be addressed?

11. What are the views and experiences of faith leaders and participants regarding the intervention?

12. What are people’s (that is, men’s, women’s and children’s) views and attitudes on the appropriateness of religious leaders taking on a health promotion role?

## Methods/design

### Study design

**Figure 1:**
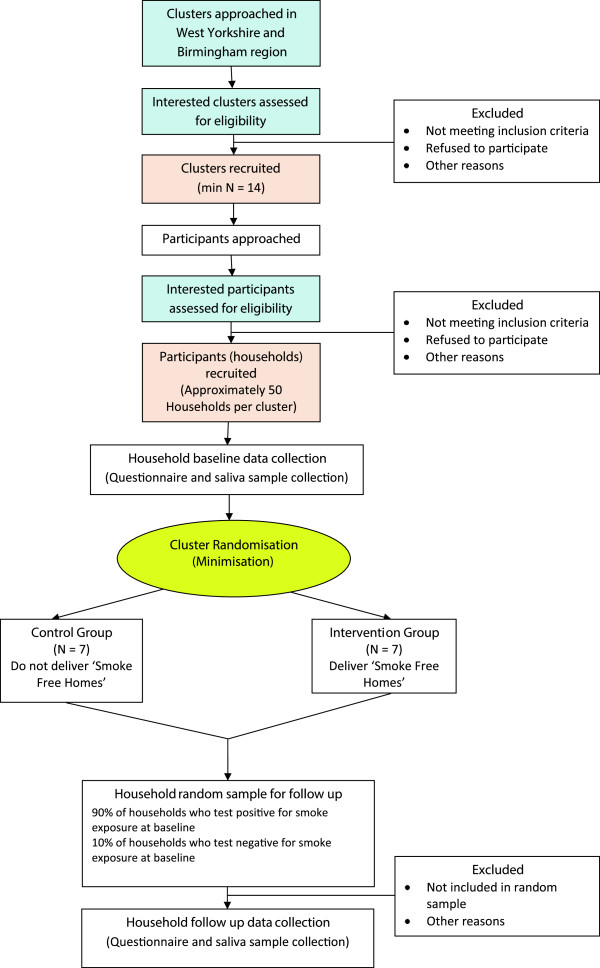
MCLASS trial flow diagram.

The design of this trial follows Phase II of the Medical Research Council’s (MRC) framework for the evaluation of complex interventions [32]. This is a pilot cluster randomised control trial (CRCT) of ‘SFH’ in Islamic religious settings with an integrated qualitative study and preliminary health economic component. The pilot trial will be carried out in approximately 14 Islamic religious settings (for example, mosques with dependent Madrassas and men’s/women’s circles, Islamic schools for children and Islamic forums) which host communal prayers, and/or convene study circles for women and/or have regular Qur’an classes for children. The clusters will be randomised to the intervention and control group in a 1:1 ratio. Clusters allocated to the intervention arm will be offered the SFH package (*Smoke Free Homes: a resource for Muslim religious teachers*) and trained to implement the package in their settings. The clusters in the control arm will not be offered the package until the completion of the study. From each cluster, we aim to recruit approximately 50 households with at least one adult resident who smokes tobacco and at least one child or a non-smoking adult resident. Households will complete a household survey and a non-smoking individual will provide a salvia sample which will be tested for cotinine. All participant outcomes will be measured before and after the intervention period in both arms of the trial. In addition a purposive sample of participants and religious leaders/teachers will take part in interviews and focus groups. Figure 1 shows the trial design and flow of participants through the trial.

### Intervention: clusters with SFH package

In the intervention group we will offer the SFH package and train faith leaders on how to use the resources. They will then implement the package in their respective settings.

The SFH pack includes:

1. Factsheets detailing key information on smoking, SHS and ‘SFH’.

2. Guidelines on how and when to use the information and/or activities.

3. Activities for different audiences:

men’s and women’s circles and Qur’an classes; discussion topics, flip chart with photos and questions, activities and role plays

children; word searches and flip charts

large groups (mixed audiences); discussion topics, flip chart, leaflet and so on.

4. Guidance and exercises on situating generic facts on smoking and second-hand smoking in an Islamic context.

5. A leaflet that contains the key facts about smoking, SHS and ‘SFH’ that can be disseminated after the Jumma khutba (Friday sermon), women’s circle meeting or to older children or parents after Qur’an classes.

These resources aim to deliver a targeted intervention for Bangladeshi- and Pakistani- origin Muslim communities to influence change in smoking behaviour. The SFH package also complies with Netto’s five established principles of planning targeted health interventions for minority ethnic communities [33].

The intervention period will last for approximately three months. Implementation will be monitored by measuring the number (and types) of activities conducted in each cluster and the approximate number of people present during each session.

### Control: clusters without SFH package

The clusters in the control group will not receive the SFH package during this pilot trial, but will be offered the SFH pack free of charge and provided with a detailed guide on how to train religious teachers on the use of the SFH pack upon completion of the trial. It is hoped that this proposal will encourage clusters to participate in the trial and reduce any disappointment at being randomised to the control group.

### Randomisation

Individual randomisation is not appropriate in this situation as it would result in contamination, since the intervention is delivered at a community/group level. We have, therefore, designed a CRCT, which is appropriate given the educational nature of the SFH intervention.

Once baseline data have been collected, participating clusters will be randomly allocated by York’s Trial Unit to one of the two trial arms, intervention or control, on an equal basis (that is, in the case of 14 clusters, seven clusters in each arm). This trial will employ restricted randomisation to avoid cluster level imbalances, using the minimisation technique for allocation, which achieves balanced groups more efficiently than other allocation methods [34]. We will minimise (that is, ensure balance) on the size of the cluster (based either on the estimated number of members or in the case of Mosques, the average size of Friday congregations), the number of consenting households for each cluster and, possibly, cluster denomination (if we recruit clusters of different denominations, for example, Shia and Sunni). Each intervention cluster will be paired with a control cluster for follow up purposes only.

### Contamination and research bias

One of the main sources of bias is contamination between the intervention and control arm due to exposure to the SFH package. By using Geographic Information System (GIS) maps, we will ensure that the catchment area of any two clusters does not overlap by aiming to have a buffer zone of at least one mile between clusters to minimise the risk of contamination [35] (unless clusters serve different denominations, in which case clusters within a one mile radius will be included).

Randomising the clusters prior to recruiting participants can also pose a risk to study validity, as knowledge of the cluster allocation may intentionally or otherwise influence who is approached, and who consents, to take part in the trial and the intervention [36]. Therefore, the recruitment of households and baseline data collection will take place prior to the randomisation of the clusters.

### Recruitment

There are two main recruitment categories: (1) Islamic religious settings (clusters) and (2) households (participants). We aim to recruit 14 clusters and approximately 50 households from each cluster.

Clusters and participants will be recruited based on the following eligibility criteria:

#### Cluster eligibility (Islamic religious settings)

Islamic religious settings (for example, mosques, madrassas, women’s/men’s circles) will be recruited from Birmingham and West Yorkshire.

#### Inclusion criteria

Participating clusters must:

1. Have a committee and an appointed Imam or faith leader

2. Be located in an area with Muslim residents of Pakistani and/or Bangladeshi ethnicity

3. Be at least one mile from another cluster. Two clusters within a mile radius of each other may be included provided they are of different denominations (Barelvi, Deobandi, Shia and so on) since they are likely to serve separate populations.

In addition, each of the clusters must fulfil at least one of the following conditions:

4. Hold regular Friday prayers with an average of at least 50 attendees; or

5. Hold regular Qur’an classes for children; or

6. Hold or be able to organise women’s/men’s circle(s).

#### Exclusion criteria

1. Have taken part in a ‘SFH’ activity before.

2. Situated within a one mile radius of an already participating cluster (unless of a different denomination).

#### Participant eligibility (households)

We aim to recruit approximately 50 households per cluster.

#### Inclusion criteria

Participating households must have:

1. An adult resident who smokes cigarettes or other form of tobacco on a regular basis.

2. A child (1- to 16-years old) or a non-smoking adult resident.

3. At least one resident who attends a participating cluster at least once a week (for example, a male adult who attends a mosque or a female adult who attends a women’s circle or a child who attends Qur’an classes).

#### Exclusion criteria

1. Any household where all adult residents smoke cigarettes or other forms of tobacco on a regular basis, and where there is no child resident.

2. There are no residents in the household who smoke on a regular basis.

3. Household members are regularly attending more than one cluster that is in the trial.

#### Cluster recruitment

We have approached the Council of Mosques in Bradford to help in the recruitment in this area. We have community links with mosques in Birmingham and West Yorkshire, including Huddersfield, Halifax, Dewsbury and Batley. We also have links with two national bodies representing mosques in the UK: Muslim Council of Britain (MCB) and UK Islamic Mission (UKIM).

Identified clusters will be contacted via chairs of their respective committees and leaders of women’s circle as appropriate to seek their interest in participating in the study. We will use our existing community links with mosque committee members and women’s circles in making the initial contact. We will visit all interested clusters and meet their committee chairs and women’s circle leaders to inform them about the study. We will explain random allocation and assure them that clusters allocated to the control arm will also be provided with the intervention after the completion of the pilot trial. Interested clusters will be provided with an invitation letter and an information sheet.

Our preliminary work suggests that mosques are usually well disposed to take part in research projects that have the potential to directly benefit the communities they serve. However, using established community networks and ensuring that early discussions happen through existing links is important in gaining their trust.

#### Cluster agreement to participate

Agreement of cluster participation will be sought from the committee chairs and/or women’s circle leaders. They will be requested to sign a written Agreement to Participate form for their cluster and themselves to take part in the study. We think this approach is reasonable given the organisational structures within mosques.

Agreement to participate will be sought for:

1. Implementing the ‘Smoke Free Homes: a toolkit for Muslim religious teachers’ (during the study in the intervention arm and after the completion of the data collection in the control arm).

2. Facilitating the research team (recruitment officers) in the recruitment of participants in respective settings.

3. Approaching committee chairs, women’s circle leaders, Imams and Qur’an class teachers to seek their consent to take part in interviews and focus groups.

4. Recording of non-identifiable mosque data according to the study protocol.

#### Household recruitment

A number of strategies will be used to recruit households/participants:

1. Recruitment stalls: These will be placed at participating clusters displaying a poster about the study, giving the option to register interest and holding information leaflets for individuals to take away.

2. Daily prayers: People who attend a mosque regularly (on a daily basis) will be invited by the Imam at the end of a daily prayer (a different prayer time will be chosen every day to approach a new set of potential participants) to attend a five minute presentation by a recruitment officer about the study. A study poster will be on display and those interested will be asked to register their interest by leaving their contact details. Attendees will also be encouraged to take the leaflet for further consideration. Contact details for the recruitment officers will also be provided should people have further questions.

3. Friday prayer/sermon: At the end of a Friday sermon (just before the Friday prayer), Imams will make a brief announcement about the study and introduce the recruitment officer. The recruitment officer will present the study to the audience (for less than five minutes) and invite people to visit the recruitment stalls after the prayers (one for men and another, where appropriate, for women). The recruitment officer will be present at the stall after Friday prayers to talk to interested people about the study. A poster and information leaflets will be displayed. The recruitment officer will also ask interested people to leave their contact details.

4. Quran classes: At the end of the Qur’an class, parents collecting their children will be invited to attend a five minute presentation about the study. A study poster will be on display and interested parents will be asked to leave their contact details. An information leaflet about the study will also be given to all parents whose children attend Qur’an classes. An information sheet for children will also be available.

5. Men and Women’s Circles: Briefings will take place at men and women’s circle meetings. The recruitment officer will talk through the study using a poster, ask interested men and women to leave their contact details and offer an information leaflet to take home for further consideration.

6. General publicity: In addition, the recruitment officer will place posters and leaflets in the mosques in visible locations and publicise the study on the local Muslim/Asian community radio and television channels.

Language and cultural barriers may impede the understanding of information pertinent to gaining informed consent from participants. In addition, lack of cultural understanding can inadvertently exclude participants, particularly South Asian people, from taking part in trials [37]. To overcome this, a comprehensive multi-approach recruitment strategy has been developed which not only supports recruitment for the MCLASS trial, but also informs members of the local community (even those not taking part) using existing networks to foster support and trust within the community. In addition, all the information sheets and consent forms will be available in the three main languages (that is, Bangla, English and Urdu) relevant to the trial participants. The research team also includes members who are proficient in English, Urdu, Mirpuri and Punjabi. Recruitment Officers will also be expected to speak at least one other relevant language, in addition to English (that is, Urdu and/or Bangla).

#### Participant screening and informed consent

As the intervention is an educational package designed to be delivered by religious leaders to all attending members of a mosque, women’s circle or Qur’an class, agreement for delivering the intervention will be sought, for example, from the mosque committee chair and/or religious leaders and not from individual participants. We think this approach is proportionate given the very low risk associated with participation and the likely potential benefits of the intervention. However, we will seek written informed consent from all participants in the household for all other research activities.

Eligible households will fall into two possible categories:

1. Households with at least one child and one adult smoker: In this case, consent will be sought from an adult resident (ideally a non-smoker) for completing the baseline (and follow-up when requested) questionnaire. A non-smoking resident (ideally a child) will be asked to consent to provide a baseline (and follow-up when requested) saliva sample. Consent will be sought from the parents/carers for a child of less than 16 years old. Children will be provided with an age appropriate information sheet and no samples will be taken if they are unwilling to participate.

2. Households with adult residents only and at least one non-smoking adult living with a smoker: Consent will be sought from the non-smoking adult resident (ideally) for completing the baseline (and follow-up when requested) questionnaire and providing a baseline (and follow-up when requested) saliva sample.

The recruitment officer will go through the respective information sheet with the potential participant during their appointment and seek consent(s) as appropriate.

We are not offering any personal incentive to the participant for taking part in the study; however, participants will be told that if they agree to participate, a sum of £5 will go towards a charity of their choice from a pre-selected list. All consents will be obtained prior to registration of participants and before any trial specific baseline assessments.

### Sample size

#### Quantitative

As this is a pilot trial whose findings will inform sample size considerations for a definitive trial, no formal sample size calculations have been undertaken. However, with the number of clusters we seek to enroll we should be able to estimate recruitment and attrition rates, effect size and ICC.

In the case of the primary outcome, the pilot trial will also provide possible effect size and standard deviation (SD) values. If these values are similar to other studies [11,38,39], we expect that the difference in the cotinine levels between people who have different levels of exposure according to the smoking restrictions at home will be more than 0.20 ng/mL. Using this, we will then plan to power the main trial to detect at least a 28% reduction in exposure to SHS. Based on an assumed standard deviation of 1.38, the estimated detectable difference of 0.20 in the mean cotinine levels between the two arms is expected. However, these assumptions are based on a different population and our study will help in testing these.

The sample size of a cluster trial depends on the number of clusters, cluster size and variation. Therefore, this study aims to determine the ICC and rates of recruitment and attrition in order to compute the design effect by which we would need to inflate the sample size if we were undertaking a fully powered individually randomised trial. We plan to recruit 14 mosques with sufficient variation to estimate ICC with some precision. Assuming that at least 50 households agree to participate from each mosque, we expect at least 20 households with a positive cotinine test as baseline. This investigation will also answer whether this is achievable. It is expected that the attrition rate will be no more than 20% for the post intervention assessment. However, actual attrition rates will aid in making adjustment to the sample size. In addition to this, these assumptions will also be tested for other outcomes.

### Outcomes

#### Pilot trial outcomes

Number of clusters (Islamic religious settings) and the size of each cluster (participants) for the main trial:

1. Recruitment and attrition rates for clusters and participants.

2. Successful recruitment strategies and barriers to recruitment.

3. Length of time required to reach participant recruitment saturation for each cluster.

4. Descriptive data on characteristics of participating clusters and participants.

5. Reasons for ineligibility of clusters and participants.

6. Reasons for willingness of clusters to be randomised.

7. Reasons for non-participation/non-consent of clusters and participants.

8. Estimate of the effect size of the primary outcome (salivary cotinine).

9. Calculation of the ICC for the primary outcome in order to inform the sample size required for a main trial.

10. Estimate of contamination between clusters.

Feasibility and resource requirements to deliver the intervention and assess its outcomes:

1. The costs associated with delivering ‘SFH’ through Islamic religious settings.

2. The feasibility and acceptability of measuring the primary outcome (salivary cotinine) including the response rate to obtaining saliva samples, and the extent and type of missing data with reasons.

3. The response rate for the household survey (including health care utilisation questions), including the extent and type of missing data with reasons.

SFH in Islamic religious settings

1. The facilitators and barriers for integration of ‘SFH’ into Islamic religious settings practice and how the facilitators might be enhanced / barriers be addressed.

2. The views and experiences of faith leaders and participants regarding the intervention.

3. People’s (that is, men’s, women’s and children’s) views and attitudes on the appropriateness of religious leaders taking on a health promotion role.

#### Definitive trial primary outcome

The primary outcome measure in a definitive trial would be salivary cotinine levels in non-smokers in the households at follow up.

#### Definitive trial secondary outcomes

Secondary outcomes in a definitive trial would be:

1. Smoking restrictions at home: We will assess the level of smoking restrictions at home through a questionnaire directed at the adults in the households.

2. Smoking status of adults and their intention to quit: We will assess the smoking status of the adults living in participating households.

3. Family health service use: We will ask about the health service use of members of the household in the three months before the intervention and in the three months during the intervention.

4. In addition, we will measure a number of other variables, which will be built into our enquiry tools for assessing the above outcomes. These will include family structure and composition (for example, number of adults and children, their age, gender, and ethnicity), socioeconomic status, employment status, number of rooms in the house and neighbourhood variables (for example, availability of smoke free environments, rural, urban, cigarette shops and so on). In addition, we will also ask about the frequency and mode of contact with the mosque, that is, prayers, study circles, Qur’an classes.

### Data Collection

#### Quantitative

##### Cluster baseline data

For each cluster in the study the following information will be recorded at the start of the study:

1. Type of cluster (ethnic and religious denominations).

2. Average estimates of people who attend two or more daily prayers.

3. Average estimate size of Friday congregation.

4. Average estimate size of study circle (men).

5. Average estimate size of study circle (women).

6. Average estimate size of Qur’an class (children).

7. Average age (self-reported by teacher) of students/children taught.

##### Survey

Approximately 50 households per cluster will be recruited for the trial. After determining household eligibility and seeking informed consent, recruitment officers will carry out a baseline household survey. This survey encompasses four main dimensions: (1) basic information about household adults and their health service usage; (2) basic information about household children and their health service usage; (3) household smoking behaviours and practices; and (4) information about the frequency and mode of contact with participating Islamic religious settings.

A follow up survey covering the same dimensions will be conducted at approximately three months post intervention start, in a selection of households who remain in the trial (a random sample of 90% of the families who test positive for exposure to SHS at baseline and a random sample of 10% of the families who test negative for exposure to SHS at baseline) in both arms of the trial.

##### Saliva

From each of the households recruited, the recruitment officers will also collect a saliva sample to measure salivary cotinine levels at baseline from a child in the house or, in the absence of a child, from a non-smoking adult member of the household. A second saliva sample will be collected from the same individual approximately three months post intervention start, in a selection of households who remain in the trial (a random sample of 90% of the families who test positive for exposure to SHS at baseline and a random sample of 10% of the families who test negative for exposure to SHS at baseline) in both arms of the trial.

Cotinine is a metabolite of nicotine that can be measured in three main forms: serum, saliva and urine. Out of the three, salivary cotinine is the most sensitive and specific measure of exposure to tobacco, with a half-life of 12 to 18 hours [40,41]. It is measured by collecting saliva in the mouth and blowing it into a plastic container through a straw or by using a swab. The samples are subsequently analysed and a gas–liquid chromatography technique can detect cotinine levels as low as 0.1 ng/ml. Based on cotinine measurements taken as part of the Health Survey for England for the years 1996 to 2004, various thresholds for active and passive smoking have been defined for different age groups [42].

#### Qualitative

Qualitative research will be conducted towards the end of this trial. The qualitative investigation will explore the facilitators and barriers for integrating ‘SFH’ into Islamic religious settings practice and how these can be enhanced or addressed; the views and experiences of faith leaders and participants regarding the intervention; and the views of participants on the acceptability of religious leaders taking on a health promotion role. This component will encompass approximately twenty to twenty-five one-to-one in-depth interviews with faith leaders (that is, mosque committee chair and religious teachers) and twelve to twenty focus group discussions with around six participants who received the SFH package. A purposive sample depending on the role of religious teachers (that is, Imam, women’s circle leader, Qur’an teacher and so on) will be selected for interview. All committee chairs in the intervention arm will also be interviewed in order to understand if and how the intervention has been delivered. Focus group discussion participants will be selected depending on the extent to which the intervention has been delivered within the cluster. The experience of the feasibility trial in Leeds, suggests that the gender distribution of participants will depend on which activities have been conducted. Mosque chairs, Imams and their participants will be men; leaders of women’s discussion groups and their participants will be women. Qur’an teachers and children may be male or female and we will sample an equal number of male and female Qur’an teachers, as far as this is possible within the parameters outlined above.

### Ethical approval

The trial has been granted ethical approval by the local NRES Committee (REC reference: 12/YH/0242) and has also been approved by the University of York, Health Sciences Research Governance Committee in May 2012.

### Data analysis

#### Quantitative

Summaries of the baseline characteristics of the clusters and households will be presented by trial arm, and recruitment and attrition rates will be reported. Although determining differences between the two arms is not the primary purpose of this study, a comparison will be undertaken to calculate an estimate for the likely effect size and ICC. Clusters will be stratified by the type of religious setting. Analysis will be conducted with the mosques as the unit of analysis using the household-level intention to treat principle (ITT).

#### Qualitative

Data collected will be transcribed verbatim and translated (as necessary). These will be organised, coded (using both *a priori* and emergent codes) and analysed thematically. *A priori* codes will be identified from relevant literature and from our previous qualitative work conducted in five mosques in Leeds, as part of the feasibility study. Taking into consideration the unique and novel setting, it is also important to explore emergent codes which will serve to explain and describe the context for a larger trial. Software (NVivo or ATLAS.ti) will be used to manage the data.

#### Economic

As this pilot trial is likely to be underpowered, we will not carry out a cost-effectiveness analysis. The health service usage questionnaire will capture participants’ utilisation of healthcare services before and after the trial intervention. We will examine any changes in service use that occurred by multiplying quantities of resources used by unit costs of healthcare. These questionnaires will be used to develop a more complete questionnaire for use in a larger trial. A larger trial will combine intervention costs with wider healthcare utilisation costs and quality of life years from EQ5D to estimate a cost-effectiveness ratio and indicate the potential value for money afforded by the intervention.

## Discussion

This is a pilot trial of ‘SFH’, an educational programme which has been adapted for use by Muslim faith leaders, in an attempt to find an innovative solution to encourage Pakistani- and Bangladeshi-origin communities to implement smoking restrictions in their homes. This pilot CRCT will establish the feasibility of conducting a definitive evaluation of ‘SFH’ for Muslim faith leaders. It will provide information to inform the design of a future definitive study. It is anticipated that results of this pilot trial will be published in summer 2014.

## Trial status

The MCLASS trial began on 1 April 2012, and will run for two years, with an expected end date of 1 April 2014. We are currently recruiting mosques and households/participants for the study.

## Abbreviations

CRCT: Cluster randomised controlled trial; CVD: Cardiovascular diseases; GIS: Geographic information system; ICC: intra-cluster correlation coefficient; ITT: intention to treat; MCB: Muslim Council of Britain; MCLASS: Muslim Communities Learning About Second-hand Smoke; MRC: Medical Research Council; NPRI: National Prevention Research Initiative; RCT: randomised controlled trial; SFH: Smoke Free Homes; SHS: second-hand smoke; UKIM: UK Islamic Mission; YTU: York Trials Unit.

## Competing interests

The authors declare that they have no competing interests.

## Authors’ contributions

KS conceived of the study and helped to prepare the manuscript. HA prepared the draft of the manuscript. FA helped to prepare the manuscript. All authors participated in study design, read and approved the final manuscript.
